# Combined Immunoscore for Prognostic Stratification of Early Stage Non-Small-Cell Lung Cancer

**DOI:** 10.3389/fonc.2020.564915

**Published:** 2020-09-25

**Authors:** Alice Boscolo, Francesco Fortarezza, Francesca Lunardi, Giovanni Comacchio, Loredana Urso, Stefano Frega, Jessica Menis, Laura Bonanno, Valentina Guarneri, Federico Rea, PierFranco Conte, Fiorella Calabrese, Giulia Pasello

**Affiliations:** ^1^Department of Surgery, Oncology and Gastroenterology, University of Padua, Padua, Italy; ^2^Pathology Unit, Department of Cardiac, Thoracic, Vascular Sciences and Public Health, University of Padua, Padua, Italy; ^3^Thoracic Surgery Unit, Department of Cardiac, Thoracic, Vascular Sciences and Public Health, University of Padua, Padua, Italy; ^4^Oncology Unit 2, Istituto Oncologico Veneto IRCCS, Padua, Italy

**Keywords:** NSCLC, immune microenvironment, early stage, prognosis, PD-L1

## Abstract

**Background:**

To date, no combined immunoscore has been evaluated for prognostic stratification of early stage non-small-cell lung cancer (NSCLC). The main goal of this study was to investigate the prognostic impact of programmed death ligand 1 (PD-L1) expression and different immune cell components (CD4+, CD8+ T-lymphocytes, and CD68+ macrophages) in early stage NSCLC patients, distinguishing peritumoral (PT) and intratumoral (IT) localizations. The secondary aim was to identify a combined immunoscore to optimize the prognostic stratification of NSCLC patients.

**Methods:**

This retrospective study included surgical specimens from consecutive chemo-naive stage II–III radically resected NSCLC patients. Immunohistochemistry was carried out to evaluate PD-L1 expression and to quantify IT and PT CD4+, CD8+ T-lymphocytes, and CD68+ macrophages. The impact of a single marker and of a combination of multiple markers on overall survival (OS) was investigated.

**Results:**

Seventy-nine patients were included in the study. PD-L1 expression was associated with worse prognosis (3 years OS: 58% in high- compared with 67% in low-expressing tumors), even though without statistical significance. When integrating PT CD8+, CD4+, and CD68 into a combined PT immunoscore, a significant prognostic stratification of patients was obtained and confirmed at multivariate analysis (3 years OS: 86% in patients with low PT immunoscore vs. 59% in patients with high PT immunoscore, *p* = 0.018). The integration of derived neutrophil/lymphocyte ratio (dNLR) with combined PT immunoscore improved prognostic stratification, with longer OS in patients with low PT immunoscore and low dNLR (*p* = 0.002).

**Conclusion:**

The combined PT immunoscore (CD8+, CD4+, and CD68) integrated with dNLR may be a promising marker for the development of an integrated Tumor, Node, Metastasis (TNM) immunoscore.

## Introduction

Lung cancer is still the leading cause of cancer-related death worldwide, and non-small-cell lung cancer (NSCLC) accounts for about 85% of cases compared with 15% of small-cell lung cancer (SCLC) (1).

Only about 20–25% of NSCLC may be considered as radically resectable and therefore potentially curable, with variable prognosis depending on the Tumor, Node, Metastasis (TNM) stage (1).

Five-year survival rate in surgical cases is improved by 4% with adjuvant chemotherapy, which currently has a role in good performance status patients with stage II and III NSCLC (2).

Histologic subtype and stage according to the TNM classification system are currently the main reference tools driving the decision-making process in these patients. However, the prognostic stratification of resected NSCLC patients should be further improved in order to optimize the selection of patients to be offered adjuvant treatments.

Immune cells within the tumor microenvironment (TME) have been shown to play an important role in the development, progression, and outcomes of NSCLC (3, 4). Some works on early stage radically resected NSCLC have previously associated tumor-infiltrating lymphocytes (TILs) and immune checkpoint expression, such as programmed death ligand 1 (PD-L1) and lymphocyte activation gene 3 (LAG-3), with patient’s prognosis (5–10). Some studies have evaluated total TILs; others have studied specific immune components, alone or in combination (8, 9, 11–14), with heterogeneous results. A recent meta-analysis focusing on the prognostic value of TME immune cells of early stage lung cancer revealed that, after adjusting for important clinical covariates (such as stage and age), higher levels of CD8+ cytotoxic T cells, CD20+ B cells, and CD 56/57+ natural killer (NK) cells seem to be associated with a better prognosis. On the contrary, lung cancers with increased FoxP3+ T regulatory cells, mast cells, and CD68+ macrophages showed worse prognosis (15). Recently, a potential positive prognostic role of tumor-associated macrophages (TAMs) has been reported (16). Concerning immune checkpoints, controversial data about the prognostic role of programmed death 1 (PD1)/PD-L1 (6, 7) and of LAG-3 expression (10, 17) are available. Additional information about the prognostic value of peripheral blood markers like neutrophil/lymphocyte ratio (NLR) in early NSCLC has been recently published (18).

The identification of an immunoscore, combining different immune cell infiltrates, appears highly promising in various tumor types, including colorectal cancer. Moreover, the inclusion of an immunoscore into the traditional TNM staging system has also been proposed for NSCLC (19). To the best of our knowledge, no standardized immunoscore combining different cell components and their spatial distribution has already been defined in NSCLC.

The primary aim of the study was to investigate PD-L1 expression and immune cell infiltrates (CD4+, CD8+ T-lymphocytes, and CD68+ macrophages), distinguishing intraepithelial [intratumoral (IT)] and peritumoral (PT) localization, in surgical resections from early stage NSCLC patients potentially eligible for adjuvant chemotherapy. The secondary aim was to identify a combined immunoscore that could optimize the prognostic stratification of NSCLC patients, especially when integrated with the TNM (i-TNM) classification system.

## Patients and Methods

### Study Design and Population

This is a retrospective, longitudinal, single-center study enrolling a consecutive series of NSCLC patients who underwent radical surgery between 2015 and 2017 at the Thoracic Surgery Unit of the University of Padua. The inclusion criteria were histological diagnosis of stage II–III NSCLC, chemo-naive patients, and adequate tissue sample obtained from radical thoracic surgery. Thus, patients treated with neoadjuvant chemotherapy and/or radiotherapy, palliative surgical procedure, or inadequate tissue block preservation were excluded.

This study was conducted according to current rules of Good Clinical Practice and principles of the Helsinki declaration. Alive patients were requested to sign the informed consent, according to Article 13 of the European Union Rule 2016/79, on personal data protection. For patients who were dead or lost to follow-up at the time of study enrollment, we used the Authorization number 9/2016 on “privacy protective rules for recording clinical data for research and study purposes.”

The following clinicopathological data were collected: gender, age, Eastern Cooperative Oncology Group (ECOG) performance status (PS), peripheral blood neutrophil and lymphocyte number, histology, disease stage according the Eighth Edition of the Union for International Cancer Control (UICC) TNM Classification of Malignant Tumors, and molecular assessment. Subsequent systemic or locoregional treatments, eventual relapse, and death were also collected. Patients were followed up with outpatient oncological visit every 3 months in the first 2 years, every 6 months until the fifth year, and every year thereafter. Brain, chest, and abdomen CT scans were performed every 6 months for the first 5 years and every year thereafter. Derived NLR (dNLR) was calculated as follows: absolute neutrophil count/(white blood cell concentration-absolute neutrophil count) (18, 20). The absolute neutrophil lymphocyte count was obtained on routine blood tests within 30 days prior to surgery.

#### Pathological Assessment and Immunohistochemistry

Primary tumor tissue specimens were consecutively collected and analyzed for the present study.

The tumors were staged according to the Eighth Edition of the UICC TNM Classification of Malignant Tumors (21) and histologically classified according to the 2015 World Health Organization guidelines on the classification of lung cancer (22).

**FIGURE 1:**
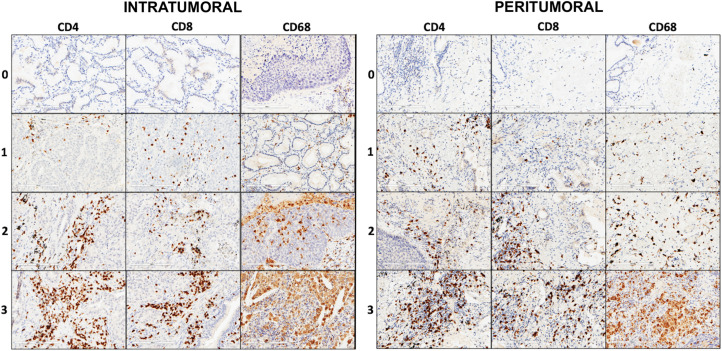
Explicative pictures of the scores (0, 1, 2, 3) for each immunohistochemistry (IHC) marker (CD4, CD8, and CD68), respectively, in IT and PT areas (IHC, original magnification × 200).

Inflammatory cell characterization was retrospectively performed by immunohistochemistry (IHC) in tissue serial sections. Briefly, 3-μm-thick sections of formalin-fixed, paraffin-embedded (FFPE) sections were mounted on glass, heated at 60°C for 20 min, and then processed using the completely automated Leica Bond III system. The slides were stained with monoclonal antibodies anti-CD8 (1:200, Dako, clone C8/144B), anti-CD4 (1:200, Dako, clone 4B12), anti-CD68 (1:200, Dako, clone PG-M1), anti-PD-L1 (1:200, Cell Signaling Tech, E1L3N). The tumor-infiltrating immune cell analysis was based on the guidelines from the International Immuno-Oncology Biomarkers Working Group (23). Briefly, the evaluation will be carried out distinguishing IT and PT compartments. IT inflammatory cells were evaluated within the borders of the invasive tumor and in areas within the tumor, considering both intraepithelial and tumoral stromal areas. Necrosis, crush artifacts, reparative fibrosis, and peribronchial sites were excluded. PT inflammatory cells were quantified in the areas around the tumor. Immunoreactivity for CD4, CD8, and CD68 was expressed as a score 0–3 (0: absent; 1: 1–33%; 2: 34–66%; 3: ≥67%) considering each marker as a percentage compared with the total amount of nucleated cells in the IT and PT compartments (24) (Figure 1). Moreover, we calculated PT/IT ratio of each inflammatory cell type to tentatively classify the tumors in “cold” and “hot” based on the prevalent PT/IT immunophenotype, taking into consideration a cutoff of 1 (lower than 1: hot; higher than or equal to 1: cold). PD-L1 expression was evaluated by counting the percentage of positive tumor cells according to the Tumor Proportion Score (TPS). Samples were considered positive when the percentage of viable tumor cells showing partial or complete membrane staining was ≥1% and distinguished in low-expression (PD-L1 < 50%) and high expression (PD-L1 ≥ 50%). Negative controls for non-specific binding were processed, omitting the primary antibodies and revealing no signal. For the assay, as a positive tissue control, normal placenta sections were used in each staining run.

Samples were anonymized and independently scored by two experienced pathologists (FC, FF).

In case of disagreement on the staining category between the two pathologists, the cases were jointly reviewed, and a single consensus category was established.

### Statistical Analysis

Statistical analysis was performed through Sigma-Plot software (Systat Software, San Jose, CA, United States) by the Kaplan–Meier estimator to evaluate and overall survival (OS). Multiple logistic regression and log-rank tests were applied. The results of the analysis were considered statistically significant when *p* < 0.05. OS was calculated as the time interval between surgery and death or last available follow-up.

Median OS and 3-year survival rates (3yOSs) were compared between the two groups defined by high and low PD-L1 expression (PD-L1 > 50% and PD-L1 < 50%, respectively) and among the four subgroups defined by different immunoreactivity scores (0–3) of CD8+, CD4+, and CD68+ cells at PT and IT level.

In order to define two main prognostic groups according to different (high vs. low) expression levels of CD8+, CD4+, and CD68+ cells, we further grouped patients with similar survival.

Cutoff expressions for CD8+, CD4+, and CD68+ cells that better stratified patient’s prognosis are summarized in Table 1.

**TABLE 1:** Definition of low and high expression of different inflammatory cell infiltrates.

Marker	Low expression	High expression
CD4+	0%	≥1%
CD8+	≤33%	>33%
CD68+	≤33%	>33%

The prognostic value of combined PT immunoscore has been corrected through COX Regression Model for other factors potentially impacting on survival: patient-related factors, such as age, gender, and adjuvant therapy; disease-related factors such as histology, lymph node involvement, and stage; and other markers like PD-L1 and dNLR.

## Results

### Study Population

Seventy-nine patients were included in the study; most of them were affected by lung adenocarcinomas (65%). The most represented stage was IIIA (49%), followed by stage II and stage IIIB (31% and 11% of the patients, respectively). About half of the patients (*N* = 36; 46%) received adjuvant chemotherapy (see Supplementary Table 1 for histologic subtype distribution and adjuvant chemotherapy by stage), whereas only 13 (16%) received adjuvant radiotherapy (50–54 Gray/27 fractions).

Baseline characteristics, immunoreactivity of each marker in eligible patients, and PT/IT cell ratio distribution across patients are summarized in Table 2. Most cases showed PT/IT CD4+, CD8+, and CD68+ ratio higher than or equal to 1.

**TABLE 2:** Patient features.

Patient features	N°	%
Total cases	79	
**Age**		
Median (y)	70	
Range (y)	39–83	
**ECOG PS**		
0	31	40%
1	48	60%
**Gender**		
Male	59	75%
Female	20	25%
**Histology**		
Adenocarcinoma	51	65%
Squamous cell ca	28	35%
**Stage TNM**		
II	31	39.2%
IIIA	39	49.4%
IIIB	9	11.4%
**Adjuvant treatment**		
Chemotherapy	37	47%
Radiotherapy	20	25%
**PD-L1 expression**		
<50%	66	84%
≥50%	12	15%
**PT-CD8+ expression**		
0%	7	9%
1–33%	14	18%
33–66%	34	43%
>66%	24	30%
**PT-CD4+ expression**		
0%	33	42%
1–33%	26	33%
33–66%	17	21%
>66%	3	4%
**PT-CD68+ expression**		
0%	2	2%
1–33%	15	19%
33–66%	25	32%
>66%	37	47%
**IT-CD8+ expression**		
0%	4	5%
1–33%	20	25%
33–66%	36	46%
>66%	19	24%
**IT-CD4+ expression**		
0%	25	32%
1–33%	34	43%
33–66%	17	21%
>66%	3	4%
**IT-CD68+ expression**		
0%	7	9%
1–33%	31	39%
33–66%	39	49%
>66%	1	1%
**PT/IT CD8+ ratio**		
<1	18	23%
>1	61	77%
**PT/IT CD4+ ratio**		
<1	27	34%
>1	52	66%
**PT/IT CD68+ ratio**		
<1	20	25%
>1	59	75%

*Ca, carcinoma; y, years; PS, performance status; IT, intratumoral; PT, peritumoral.*

At the data cutoff of November 4, 2019, median follow-up was 38 months.

**FIGURE 2:**
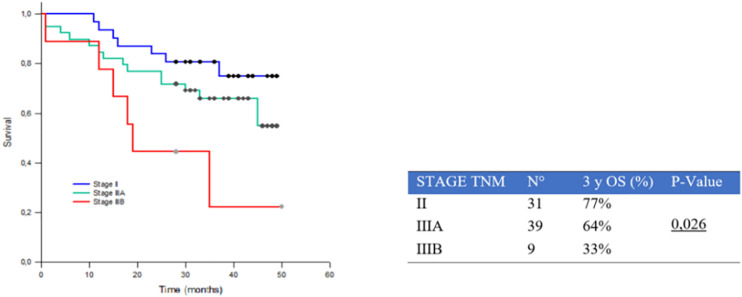
Three-year survival rate (3yOS) data and curves according to II, IIIA, and IIIB Tumor, Node, Metastasis (TNM)-pStage.

Median OS in the study population was 3 years. The 3yOSs were 77% for stage II, 64% for stage IIIA, and 33% for stage IIIB patients (*p* = 0.026) (Figure 2).

### Prognostic Impact of Different Inflammatory Cell Populations, Programmed Death Ligand 1 Expression and Integration With Tumor, Node, Metastasis Stage Classification

**FIGURE 3:**
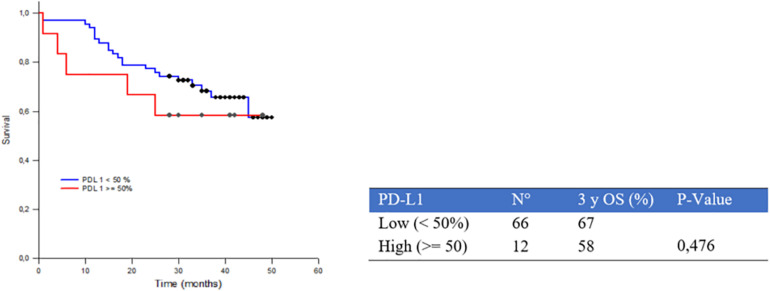
Three-year survival rate (3yOS) data and curves according to low and high programmed death ligand 1 (PD-L1) Tumor Proportion Score (TPS) expression.

High PD-L1 expression showed an association trend with a worse prognosis, even though without statistical significance (*p* = 0.476) (Figure 3).

As far as inflammatory cell infiltration is concerned, we did not observe a significant difference between groups according to IT inflammatory infiltrate (Supplementary Figure 1), thus we focused survival analyses on PT score on the basis of the better prognostic stratification.

**FIGURE 4:**
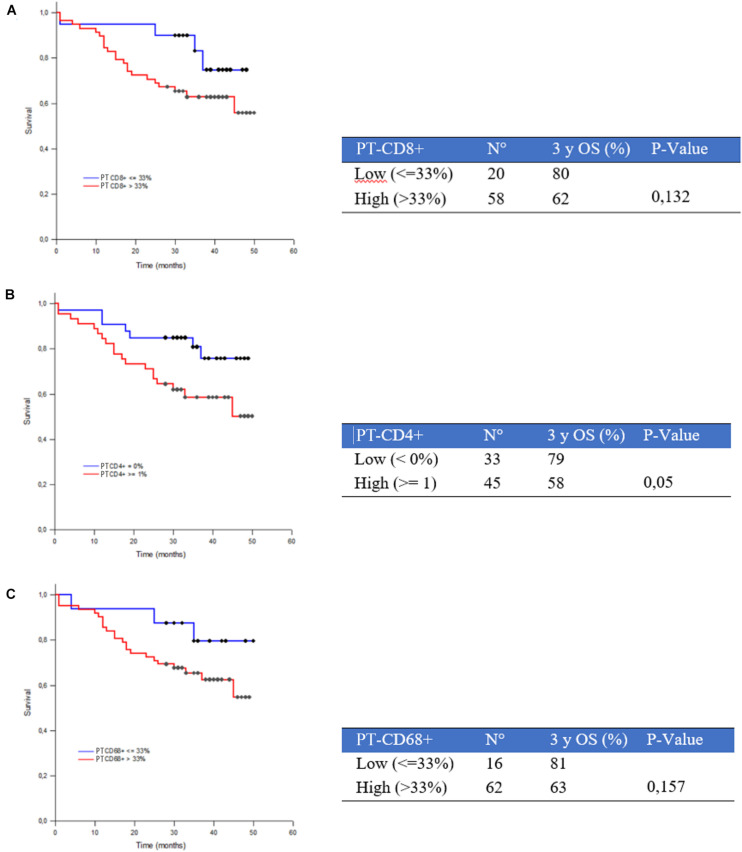
Three-year survival rate (3yOS) data and curves according to low and high peritumoral density of CD8+ T-lymphocytes **(A)**, CD4+ T-lymphocytes **(B)**, and CD68+ macrophages **(C)**.

The 3yOSs in high and low PT CD8+ subgroups were 62% and 80% (*p* = 0.132), in high and low PT CD4+ subgroups were 58% and 79% (*p* = 0.05), in high and low PT CD68+ subgroups were 63% and 81% (*p* = 0.157), respectively, (Figure 4). We also investigated survival differences between cases with CD4+, CD8+, and CD68+ PT/IT ratio lower than 1 and cases with PT/IT ratio higher than or equal to 1; although without statistical significance, we observed a trend toward longer survival for cases with PT/IT ratio lower than 1 (named “hot” tumor immune microenvironment) (Supplementary Figure 2).

**FIGURE 5:**
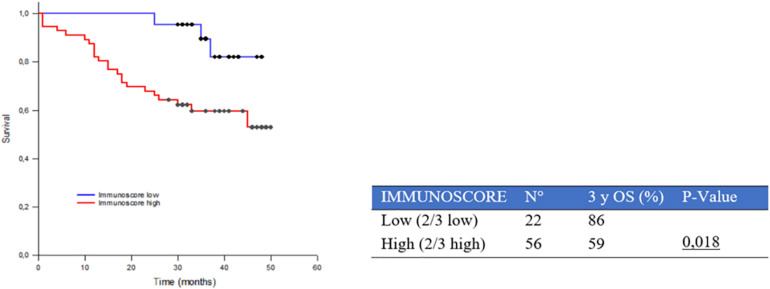
Three-year survival rate (3yOS) data and curves according to low and high combined peritumoral (PT) immunoscore. The immunoscore is given by the combination of different peritumoral inflammatory components (PT CD8+, PT CD4+, and PT CD68+).

In order to improve the prognostic stratification of the study population, we combined the different stromal inflammatory components (PT CD8+, S CD4+, S CD68+) into a combined PT immunoscore characterized by two groups: high (when at least two of the three inflammatory components were highly expressed) and low (when at least two of the three inflammatory components showed a low expression). When we compared OS curves between these two groups, we showed a worse prognosis in those cases with high compared with low combined PT immunoscore: 3yOS was 59% and 86% in the two groups, respectively, (*p* = 0.018) (Figure 5).

Furthermore, we evaluated the prognostic impact of the combined PT immunoscore within different pathologic stages showing a significant difference between low and high groups in particular in stage IIIA (93% and 48% 3yOS, respectively, *p* = 0.009) (Table 3 and Supplementary Figure 3).

**TABLE 3:** Three-year survival of stage II, IIIA, and IIIB non-small-cell lung cancer (NSCLC) patients according to high or low combined peritumoral (PT) immunoscore.

Stage	Combined PT Immunoscore	N°	3yOS (%)	*P*-Value
II	Low	**7**	**86%**	0.614
	High	23	**78%**	
IIIA	Low	14	**93%**	0.009
	High	25	**48%**	
IIIB	Low	1	**0%**	0.793
	High	8	**38%**	

Finally, Table 4 highlights 3yOS of patients according to combined PT immunoscore and TNM-pStage in the integrated TNM-PT immunoscore, showing greater accuracy in prognostic stratification of resected NSCLC when different predictors are combined.

**TABLE 4:** Three-year survival rates in different subgroups defined by the integration of combined immunoscore with Tumor, Node, Metastasis (TNM) stage classification: On the first column, 3-year survival rates according to stages I, II, and III are reported.

3-year survival rate (%)	Combined immunoscore (PT CD8+, CD4+, CD68+)	
pStage	Low 86%	High 59%
II 77%	86%	78%
IIIA 64%	**93%**	**48%**
IIIB 43%	0%	38%

*In the second and third columns, 3-year survival rates of low and high peritumoral (PT) combined immunoscore within each stage are reported. In bold, statistically significant different values are reported. Low PT combined immunoscore stage IIIB was present in one patient.*

### Prognostic Impact of Derived Neutrophil/Lymphocyte Ratio

**FIGURE 6:**
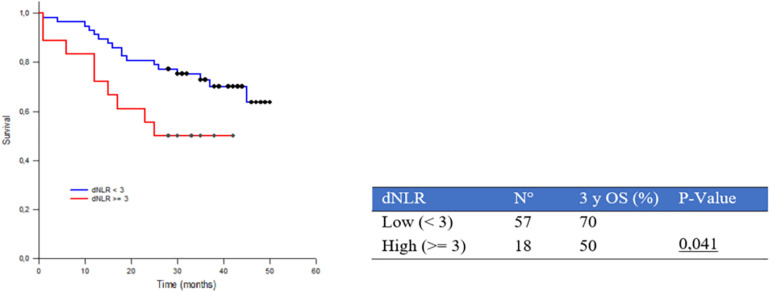
Three-year survival rate (3yOS) data and curves according to low and high derived neutrophil/lymphocyte ratio (dNLR) [absolute neutrophil count/(white blood cell concentration-absolute neutrophil count)].

We also evaluated the potential prognostic value of dNLR (using the standard cutoff of 3) (18). The results highlight a significant correlation between dNLR and survival: 3yOS was 50% and 70% in high (≥ 3) and low (<3) dNLR, respectively, (*p* = 0.041) (Figure 6).

On the basis of these data, we assessed the prognostic impact of the integration of dNLR with combined PT immunoscore, thus defining three groups: high dNLR and high PT immunoscore; high dNLR and low PT immunoscore or low dNLR and high PT immunoscore; and low dNLR and low PT immunoscore.

**FIGURE 7:**
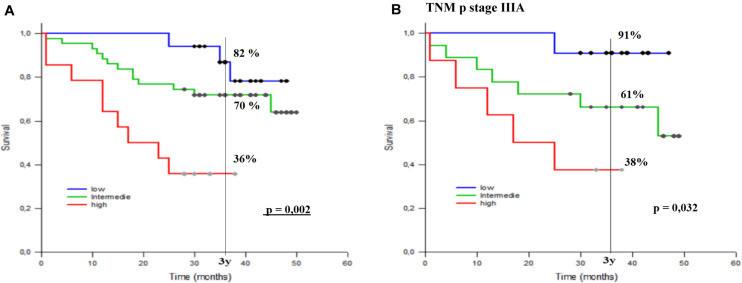
Three-year survival rate (3yOS) data and curves according to low, intermediate, and high combined peritumoral (PT) immunoscore integrated with derived neutrophil/lymphocyte ratio (dNLR) [absolute neutrophil count/(white blood cell concentration - absolute neutrophil count)] in all patients **(A)** and in those with pStage IIIA **(B)**. The combined PT immunoscore is given by the combination of different PT inflammatory components (PT CD8+, S CD4+, S CD68+).

We showed an improvement of prognostic stratification, with longer survival in patients with combined low PT immunoscore and low dNLR (*p* = 0.002) (Figure 7A); patient stratification was even better in stage IIIA (Figure 7B).

The multivariate analysis showed that the combined PT immunoscore is an independent negative prognostic factor (*p* = 0.048) with HR 0.255 (95% CI 0.0656–0.989) (Table 5).

**TABLE 5:** Multivariate analysis showing the impact of different covariates on overall survival.

Covariate	Variables	*P* Value
PD-L1	<50%; >=50%	0.575
***PT- Immunoscore***	***low; high***	***0.048***
dNLR	<3; >=3	0.204
Stadio	II; IIIB	0.076
Stadio	IIIA; IIIB	0.133
Histology	Adeno; Squamous	0.175
Limph node	+; −	0.104
Adjuvant CT	Yes; No	0.519
Gender	M; F	0.185
Age (y)	<70; >=70	0.848

*Statistical significance is indicated in bold.*

## Discussion

Surgically resected early stage NSCLC patients have a variable recurrence rate according to pathological (p) TNM stage (1). Limited weapons are currently available in the clinical practice to improve survival rates of resected patients; among these, platinum-based chemotherapy of pathological stage II and III patients improves 5-year survival rates of 4% (2), with some toxicities to be considered by the medical oncologist in order to maintain an acceptable risk/benefit ratio.

For this reason, based on the good results shown in the metastatic setting, several clinical trials with immune checkpoint inhibitors (ICIs) have been recently launched in the adjuvant setting to evaluate their potential impact on the recurrence risk reduction (25). In this context, the importance of identifying an immune prognostic predictor to be integrated with the classic TNM stage classification has recently emerged to improve risk stratification and patient selection for adjuvant systemic treatment.

Few data are available on the prognostic value of multiple immune components. Teng et al. (14) evaluated CD8+ T-lymphocytes in association with Foxp3+ T-Reg lymphocytes, concluding for a potential positive prognostic role of high levels of intratumoral CD8+ T-lymphocytes. Hald et al. (26) pointed out that the high co-expression of CD4+ and CD8+ T-lymphocytes in the tumor stroma correlated with improved survival.

While these data refer to IT immune infiltrate, we investigated tumor immune microenvironment as a whole both at the PT and IT levels. The main innovative feature of our study is the evidence that high peritumoral levels of both T cells (CD4+, CD8+) and macrophages (CD68+) were associated with a worse prognosis. The same trend has been maintained across different TNM pStages, and in the largest subgroup, stage IIIA, the prognostic impact was significant.

The biological rationale of this correlation can be explained since more aggressive tumors with a worse prognosis are more impenetrable to the inflammatory infiltrate that accumulates at the stromal level. This would be the case, under the theory by Chen and Mellman (27), of tumors with an immune exclusion phenotype, also called “cold tumors,” in which the antitumor response has been made ineffective by a blockage of penetration into the tumor, for which it is characterized by abundant external immune cells and few or absent inside. The high level of cytotoxic (CD8+) T-lymphocytes, associated with a poor infiltration of CD8+ and the lack of their activation marker expression (B2MP and IFN6), is a characteristic indicative of immunological ignorance, an immunological state in which adaptive immunity is unable to recognize or respond to a pathogen or malignancy (28).

Tumors with extensive IT inflammation are considered immunologically “hot tumors,” characterized by the presence of a large IT cytotoxic T-lymphocyte expressing PD-1, leukocytes, and tumors expressing the immune dampening PD-1 ligand PD-L1. In the latter, it seems that ICIs are more effective (27).

No correlation has been shown between the IT infiltration of CD8+ and CD4+ T-lymphocytes and CD68+ macrophages and survival. These data are in contrast with the literature and in particular with the large study on 797 NSCLC patients conducted by Donnem et al. (11, 19) where IT CD8+ lymphocytes correlated positively with survival and this correlation remained significant across different stages (26). In the same way, we could not confirm the negative prognostic value of high PD-L1-expressing tumors. That is likely due to the limits of our study, which lie in its retrospective nature and limited number of patients.

Prognostic stratification was improved by the whole assessment of different PT infiltrates within a combined immunoscore, which has been confirmed as an independent prognostic factor at the multivariate analysis. When the PT combined immunoscore was integrated with dNLR, the prognostic stratification is further improved, and this suggests the importance of integrating inflammatory biomarkers, both in tumor tissue and blood, in survival prediction.

On the basis of these preliminary data, it is tempting to speculate that PT combined immunoscore may be a promising marker for the development of an integrated TNM immunoscore, though a validation study on a prospective wider series is needed. Moreover, following up prospectively our case series, we can confirm a prognostic role of the inflammatory component in early stage NSCLC and overall validate the predictive role of cold and hot tumor immune microenvironment in advanced/metastatic NSCLC patients treated with single-agent ICI.

## Data Availability Statement

The raw data supporting the conclusions of this article will be made available by the authors, without undue reservation.

## Ethics Statement

The studies involving human participants were reviewed and approved by the Comitato Etico Istituto Oncologico Veneto. The patients/participants provided their written informed consent to participate in this study.

## Author Contributions

AB, GP, and FC contributed to the study design. AB, GP, GC, SF, JM, LB, FR, VG, and PC contributed to patient recruitment. FF and FC contributed to sample collection and pathological analyses. AB, GP, and LU contributed to data collection and statistical analysis. AB, GP, FC, and VG contributed to manuscript writing. All authors contributed to the article and approved the submitted version.

## Conflict of Interest

The authors declare that the research was conducted in the absence of any commercial or financial relationships that could be construed as a potential conflict of interest.
