# Supplementary material for: Combined Immunoscore for Prognostic Stratification of Early Stage Non-Small-Cell Lung Cancer
Source: Front Oncol. 2020 Sep 25;10:564915. doi: 10.3389/fonc.2020.564915 (PMC7544833; doi:10.3389/fonc.2020.564915)
Supplement: Supplementary file 1 [file Data_Sheet_1.docx]

Supplementary Material

1 Supplementary Figures and Tables

**1.1 Supplementary figures**

**Supplementary figure 1**.

3y OS curves according to 0%, 1-33%, 33-66% and > 66% density of intratumoral CD8+ T-cell (A), CD4+ T-cell (B) and CD68+ macrophage cell (C).

**Supplementary figure 2**

Survival curves according to CD8+ (A) CD4+ (B) CD68+ (C) peritumoral/intratumoral ratio lower than1 and higher than or equal than 1. 3y OS rates are also reported in the table.

**Supplementary figure 3**

3y OS curves according to II, IIIA and IIIB TNM-pStage in all patients (A) and 3y OS curves according to low and high combined PT-immunoscore in patients with II (B), IIIA (C) and IIIB (D) TNM pStage. The PT-immunoscore is given by the combination of different stromal inflammatory components (PT CD8+, PT CD4+, PT CD68+).

- 1. **Supplementary tables**

**Supplementary table 1**. Histologic subtypes and systemic treatment (adjuvant and palliative) distribution across stages.

| STAGE  (TNM, VIII ed) | N (%) | Adeno  N (%) | Squamous  N (%) | Adj-CT  N (% ) | Adj-RT  N (% ) | 1L-Tp  N (%) | > 1L-Tp  N (%) |
| --- | --- | --- | --- | --- | --- | --- | --- |
| II | 31 (39.2) | 22 (71) | 9 (29) | 13 (42) | 0 | 3 (9.7) | 3 (9.7) |
|  |  |  |  | Cis-Gem 6 (46) |  | CT 2 (67) | CT 2 (67) |
|  |  |  |  | Cis-Vino 1 (8) |  | Immuno 1 (33) | Immuno 1 (33) |
|  |  |  |  | Not known 6 (46) |  |  |  |
| IIIA | 39 (49.4) | 23 (59) | 16 (41) | 17 (44) | 9 (23) | 8 (21) | 3 (7.7) |
|  |  |  |  | Cis-Gem 9 (53) |  | CT 4 (50) | CT 2 (67) |
|  |  |  |  | Cis-Vino 2 (11) |  | Immuno 1 (13) | Immuno 1 (33) |
|  |  |  |  | Not known 6 (33) |  | TKI 1 (13) |  |
|  |  |  |  |  |  | Not known 2 (25) |  |
| IIIB | 9 (11.4) | 6 (67) | 3 (33) | 6 (67) | 4 (44) | 3 (33) | \ |
|  |  |  |  | Cis-Gem 2 (33) |  | Immuno 2 (67) | \ |
|  |  |  |  | Cis-Pem 2 (33) |  | Not known 1 (33) | \ |
|  |  |  |  | Not known 2 (33) |  |  |  |
|  |  |  |  |  |  |  |  |

Ed: edition; Adj-CT: adjuvant chemotherapy; Adj-RT: adjuvant radiotherapy; 1L-Tp: first line therapy for relapsed disease; >1L-Tp: systemic treatment beyond first line for relapsed disease; CT: chemotherapy; Immuno: Immunotherapy; TKI: tyrosine kinase inhibitor; Cis-Gem: cisplatin-gemcitabine; Cis-Vino: cisplatin-vinorelbine; Cis-Pem: cisplatin-pemetrexed.
