# Supplementary figures and images for: Combined Immunoscore for Prognostic Stratification of Early Stage Non-Small-Cell Lung Cancer
Source: Front Oncol. 2020 Sep 25;10:564915. doi: 10.3389/fonc.2020.564915 (PMC7544833; doi:10.3389/fonc.2020.564915)

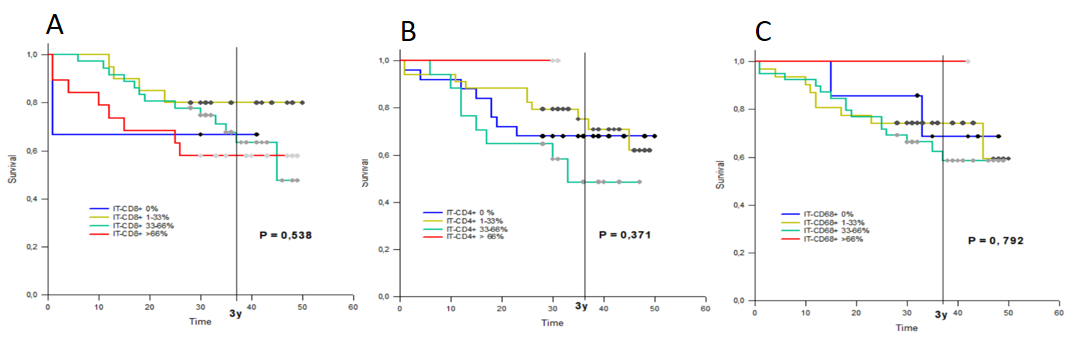

Supplement: Supplementary file 2 [file Image_1.TIF]

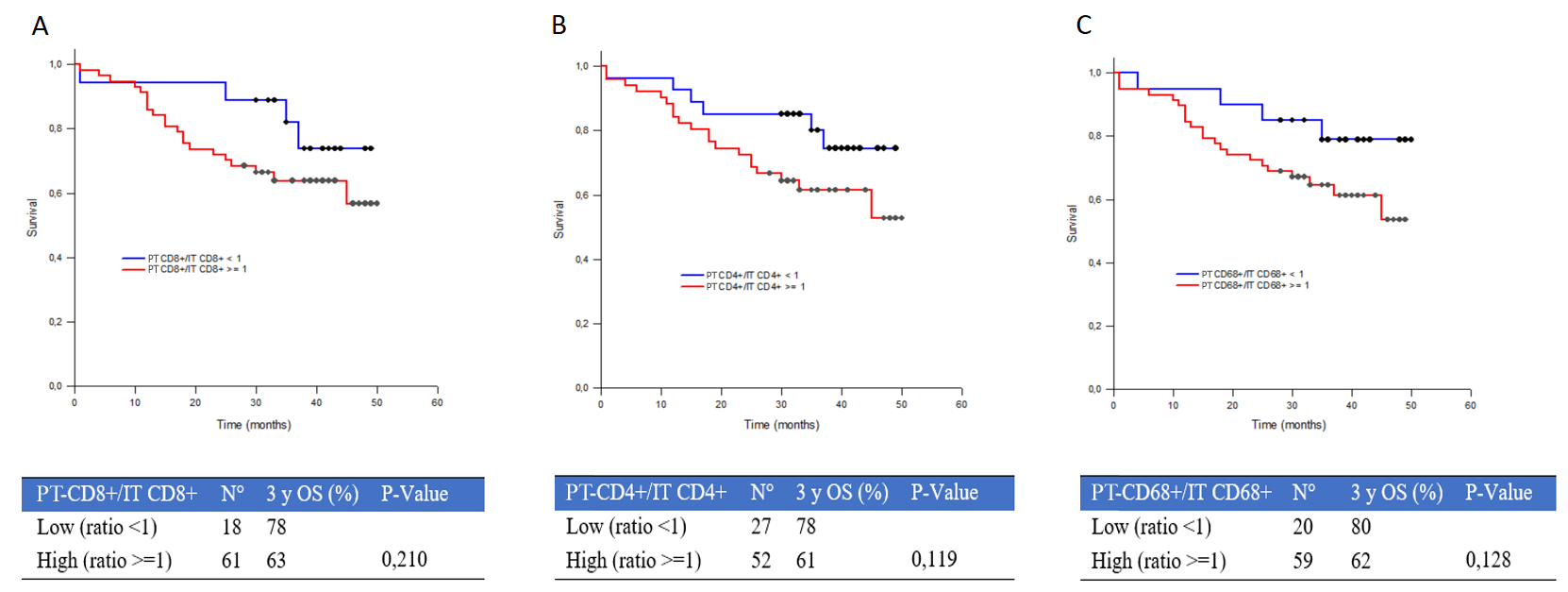

Supplement: Supplementary file 3 [file Image_2.TIF]

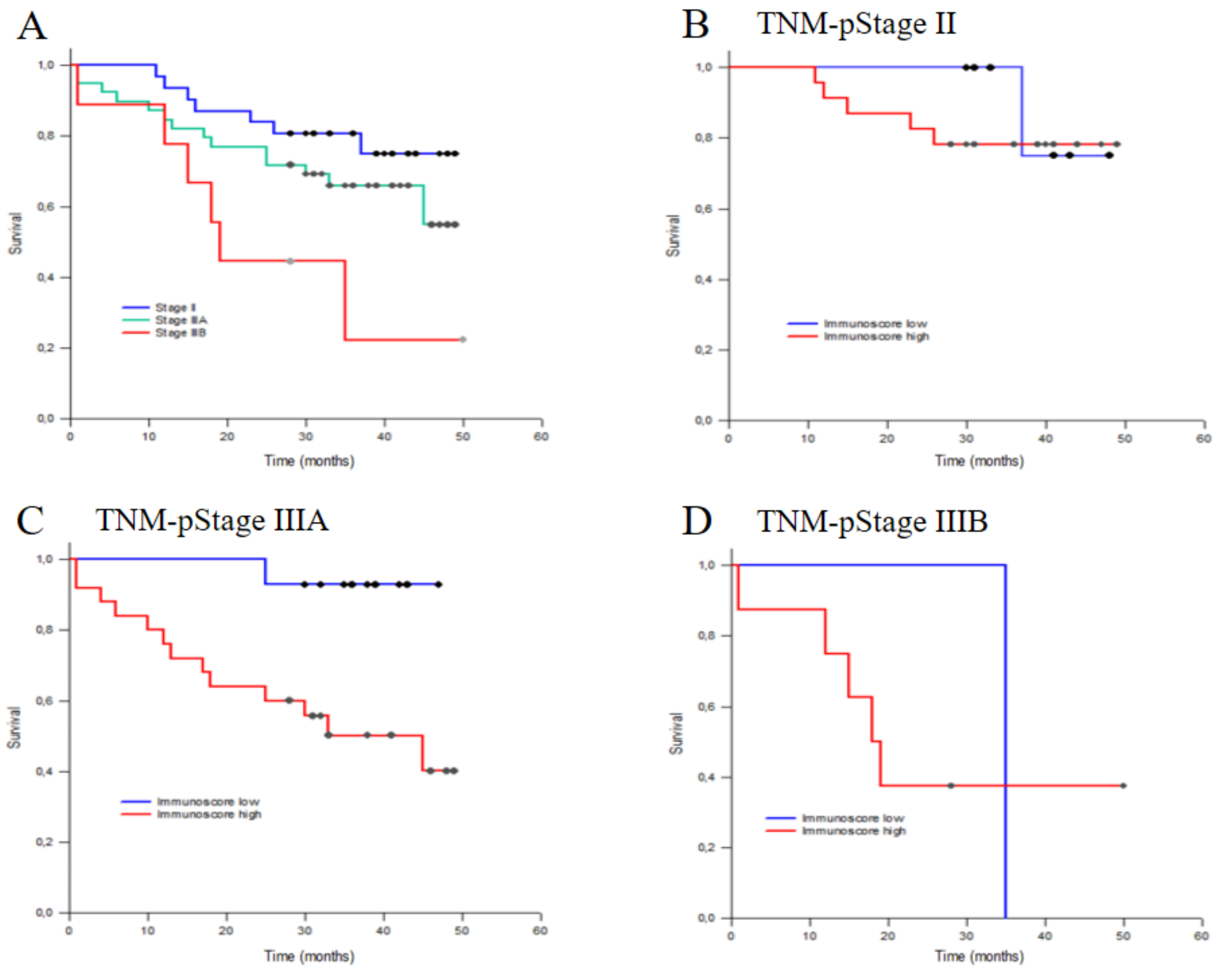

Supplement: Supplementary file 4 [file Image_3.TIF]
